# Rapid and simultaneous determination of mixed pesticide residues in apple using SERS coupled with multivariate analysis

**DOI:** 10.1016/j.fochx.2024.101954

**Published:** 2024-11-01

**Authors:** Ting-feng Shi, Ting-tiao Pan, Ping Lu

**Affiliations:** State Key Laboratory of Green Pesticide, Key Laboratory of Green Pesticide and Agricultural Bioengineering, Ministry of Education, Center for R&D of Fine Chemicals of Guizhou University, Guiyang 550025, China

**Keywords:** Simultaneous detection, Multi-residue analysis, Chemometrics, Multivariate analysis, SERS

## Abstract

This study aims to apply multivariate analysis algorithms for modeling the same spectra, for simultaneous determination of pymetrozine and carbendazim residues in apple. To mitigate the impact of competitive adsorption, SERS spectra are obtained from mixed solutions of pymetrozine and carbendazim at varying concentration ratios, which are then utilized for modeling. Results suggest that the PLSR model based on full-band SNV processed spectra shows the best performance for predicting pymetrozine and carbendazim contents, with R2 *p* of 0.9751 and 0.9779, RMSEP of 0.0492 and 0.5531 mg/L, RPD of 6.4297 and 6.8246, respectively. This model yielded R2 *p* of 0.9644 and 0.9671, RMSEP of 0.0747 and 0.8247 mg/L, RPD of 5.3857 and 5.6066 for pymetrozine and carbendazim in apple, respectively. The findings suggest that the proposed approach is suitable for simultaneous detection of pymetrozine and carbendazim in apples, offering a novel avenue for monitoring food safety.

## Highlights

• SERS was used to simultaneously detect pymetrozine and carbendazim residues in apple.
• SERS intensity of pesticides changed obviously due to competitive adsorption.
• Spectra of mixed solution with different concentration ratios were used for modeling.
• PLSR model showed superior result for pymetrozine and carbendazim content prediction.
• All models exhibited the best result by using the SNV processed spectra for modeling.

## 1 Introduction

Pesticides play a vital role in modern agriculture for their efficient improvement of agricultural production by protecting crops from diseases, pests, insects, and weeds (Huang, Song, Li, Mu, & Liu, 2019). However, with the increasing variety and amount of chemical pesticides employed in agricultural production, multiple pesticide residues are often present in a single sample, the potential hazard of pesticide residue to the environment and human health is rising (Mostafalou & Abdollahi, 2013). Given the extensive use of pesticides on a global scale, it is crucial to consistently monitor pesticide residues.

Chromatographic methods such as gas chromatography (GC), high-performance liquid chromatography (HPLC), GC coupled with mass spectrometry (GC–MS), and liquid chromatography coupled with tandem mass spectrometry (LC-MS/MS) have often been employed to detect multiple pesticide residues (Harischandra et al., 2021). These methods are sensitive, accurate, reliable, and well selective. However, their disadvantages include long experimental time, complex sample pretreatment, the need for trained personnel, and high costs. Hence, there is a demand for developing simple, rapid, in-situ, and cost-effective methods to detect multiple pesticide residues. At present, lots of simple and rapid techniques such as spectroscopy, electrochemistry, and biosensors have been developed for the detection of multiple pesticide residues (Umapathi, Ghoreishian, Sonwal, Rani, & Huh, 2022; Zhang et al., 2021). However, these methods have many shortcomings, e.g., the electrochemical methods usually require specialized workstations and electrodes, and the immunoassay method based on test strips needs expensive and unstable antibodies, and often false negative results appear (Wang et al., 2022). Due to its merits of high sensitivity, quick response, and molecule fingerprint specificity (Jiang et al., 2021), surface-enhanced Raman scattering (SERS) technique is widely used to detect pesticide residues in food and agricultural product (Chen et al., 2020; Zhu, Xu, Ali, Ouyang, & Chen, 2021). In addition, SERS is expected to be one of the best methods for in-situ and on-site detection of pesticide residues due to the acquisition of SERS spectrum is a nondestructive and rapid procedure. However, most of these studies have focused on the determination of single pesticide residues, which fall short of adequately meeting an important need of food industry: simultaneous detection of multiple pesticide residues in foodstuff.

The distinctive peaks of various analytes enable their discrimination within a mixture, facilitating the identification and detection of multiple analytes through a single SERS analysis (Wu et al., 2013). Hence, SERS is considered to be an optional method for simultaneous identification and quantification of multiple pesticides (Zhang et al., 2014). To date, however, SERS technique for simultaneous quantitative analysis of multiple pesticides in food sample has rarely been reported (Li et al., 2022; Ma et al., 2023; Yang et al., 2022). An important reason is the competitive adsorption of multiple pesticides on metal nanoparticle surface, resulting in a significant change in SERS intensity compared with the presence of one pesticide alone, which makes accurate quantitative analysis of these pesticides very difficult (Ma et al., 2023). Multivariate analysis is a powerful mathematical tool to use spectral data to construct a prediction model for qualitative and quantitative analysis of analyte in the unknown sample (Janci et al., 2017; Li, Chen, Zhao, & Wu, 2015). In addition, multivariate analysis offers the potential to utilize full-band spectra or variables associated with the target analyte, rather than relying solely on a single characteristic peak (Xu et al., 2021). Therefore, multivariate analysis is considered as an alternative way to solve the competitive adsorption problem. By integrating the advantages of multivariate analysis, the SERS technique can effectively improve its quantitative analysis ability and constantly expand its application prospect in practical detection. To date, the utilization of SERS technology in conjunction with multivariate analysis for the simultaneous detection of pymetrozine and carbendazim has not been documented.

In the present study, the potential of SERS coupled with chemometric methods for simultaneous determination of pymetrozine and carbendazim residues in apple was investigated. To reduce the effect of competitive adsorption of pymetrozine and carbendazim on Au@AgNPs surface and improve the accuracy and robustness of the built model, a series of spectra of the mixed solutions with various concentration ratios were first collected and used for modeling. To select a suitable model, different multivariate analysis algorithms were applied to construct the prediction models. Moreover, to improve the predictive stability, several spectral preprocessing methods were applied to process the raw spectra before modeling. In addition, the predicted results based on the full-band spectra and optimal variables were also compared.

## 2 Material and methods

### 2.1 Chemicals and materials

Pymetrozine standard (C_10_H_11_N_5_O), carbendazim standard (C_9_H_9_N_3_O_2_), chloroauric acid (HAuCl_4_•3H_2_O), trisodium citrate (Na_3_C_6_H_5_O_7_), silver nitrate (AgNO_3_), ascorbic acid, and sodium hydroxide (NaOH) were bought from Aladdin Reagent (Shanghai) Co. Ltd. (Shanghai, China). Sodium chloride (NaCl), anhydrous magnesium sulfate (MgSO_4_), primary secondary amine (PSA) sorbent, anhydrous methanol (CH_3_OH), ammonium hydroxide (NH_3_•H_2_O), and acetonitrile (HCN) were purchased from Chongqing Southwest Chemical Reagent Co. Ltd. (Chongqing, China). C_18_ sorbent was provided by Agela Technologies (Tianjin, China). Ultrapure water was prepared by the Millipore Milli-Q system. All glassware and magnetic stirrers were soaked in aqua regia [HCl: HNO_3_ = 3:1 (*v*/*v*)] for 24 h and washed with ultrapure water before use. All chemical reagents used in the current study were of analytical grade and used without further purification.

### 2.2 Preparation and characterization of Au@AgNPs

Au@AgNPs were prepared by seed growth method according to the previous report (Guo et al., 2022). The effects of sodium citrate concentration on the synthesis of gold core and the dosages of ascorbic acid and AgNO_3_ on the formation of silver shell were evaluated in this study. In brief, the gold core was first prepared by the sodium citrate reduction method (Frens, 1973). Then, the silver shell was prepared by seed-mediated method. The details are as follows, 500 μL HAuCl_4_·3H_2_O solution (10 g/L) was added into a round bottom flask (150-mL) containing 60 mL deionized water under continuous stirring, and the HAuCl_4_ solution was heated to boil and kept at 115 °C for 5 min. After that, different volume (400, 600, 800, 1000, and 1200 μL) of trisodium citrate solution (1 %, *m*/*v*) was quickly dropped into the above solution at one time. The resultant solution was kept stirring and reacted fully for 20 min at 115 °C. The newly prepared AuNPs colloid was naturally cooled to 25 °C at room temperature for further use. The preparation steps of the silver shell are as follows. Firstly, varying dosages (150, 300, 450, 600, and 750 μL) of 10 mM ascorbic acid was added into 9 mL of the prepared AuNPs seeds in a round bottom flask (25-mL) with vigorous stirring for 2 min, and then the corresponding volume (120, 240, 360, 480, and 600 μL) of 10 mM AgNO_3_ solution was added dropwise to the above solution. The resultant solution was stirred vigorously and reacted fully for 20 min at 25 °C. UV–vis spectra and colors of the newly prepared AuNPs and Au@AgNPs are given in Fig. S1. With increasing the dosage of trisodium citrate, the diameter of AuNPs gradually decreased judging by the blue shift of the absorption peak, and the color of AuNPs colloid changed from faint purple to wine-red to apricot pink. The Ag shell thickness was increased with increasing the amounts of ascorbic acid and AgNO_3_ based on the intensity characteristic plasmonic properties of Ag shell was increased and tended to red shift. The color of Au@AgNPs colloid changed from red to yellow to orange yellow. According to the UV–vis spectra, colloidal colors, and a previous study, the amount of trisodium citrate, ascorbic acid, and AgNO_3_ was determined to be 800, 450, and 600 μL, respectively (Wang et al., 2019).

The optical properties of Au@AgNPs and AuNPs were measured by a UV–vis spectrophotometer (TU-1901, Beijing Purkinje General Instrument Co., Ltd., Beijing, China). The size distributions of AuNPs and Au@AgNPs were investigated by dynamic light scattering (DLS) analysis using a laser light scatterer (BI-200SM, Brookhaven Instruments Corporation, New York, USA). The morphology and composition of Au@AgNPs were characterized by a transmission electron microscope (TEM), high-angle annular dark-field scanning transmission electron microscopy (HAADF-STEM), and energy-dispersive X-ray spectroscopy (EDS) (JEM-1200, JEOL Ltd., Tokyo, Japan).

### 2.3 Preparation of pesticide standard solutions and mixed pesticide solutions

A stock solution of pymetrozine in a concentration of 100 mg/L was prepared by dissolving the pymetrozine standard in methanol, and the same method was used to prepare the carbendazim stock solution (100 mg/L). Pymetrozine standard solution with different concentrations of 1.0, 0.5, 0.25, 0.1, 0.05, 0.025, and 0.01 mg/L and carbendazim standard solution with different concentrations of 10.0, 5.0, 2.5, 1.0, 0.5, and 0.1 mg/L for SERS determination was prepared by diluting the pymetrozine and carbendazim stock solutions with ultrapure water, respectively.

The mixed solutions of pymetrozine and carbendazim with different concentration ratios were prepared by mixing both 100 μL of the pymetrozine standard solution and 100 μL of the carbendazim standard solution with a concentration of twice their required concentration. Finally, the mixed solutions with totals of 32 concentration ratios were prepared according to Table S1.

### 2.4 Sample preparation

Organic apple was purchased from a local supermarket in Guiyang City and used as a representative food sample to evaluate the feasibility of the method for simultaneous detection of pymetrozine and carbendazim residues in real food samples.

Apple is a complex matrix because it contains various components, such as sugars and organic acids. These components can affect the SERS signal of pesticides during the spectral acquisition process. Thus, an appropriate pretreatment method is needed to eliminate these interfering components. Pymetrozine and carbendazim in the spiked apple samples were extracted by a modified QuEChERS method involving several main steps: sample grinding, acetonitrile extraction and separation of pesticide, removal of water using MgSO_4_ and other salts, and elimination of interference from organic acids, fatty acids, and carbohydrates in the fruit samples using PSA, C_18_ and other adsorbents (Anastassiades, Lehotay, Stajnbaher, & Schenck, 2003; Pan, Guo, Guo, Lu, & Hu, 2021). Briefly, the spiked apple samples with 6 spiked levels of pymetrozine and carbendazim were prepared by adding 2.0 mL of the pymetrozine and carbendazim mixed solution (the concentration of pymetrozine and carbendazim in level 1 to 6 was 1.0 and 1.0, 1.0 and 2.0, 0.7 and 5.0, 0.3 and 10.0, 0.1 and 12.0, 0.05 mg/L and 12.0 mg/L, respectively) into a centrifuge tube (50-mL) with 5.0 g of the homogenized apple samples. Then, 18.0 mL acetonitrile (with 1 % acetic acid) was added to the above tube for extraction, and 3.0 g MgSO_4_ plus 3.0 g NaCl were added for liquid-liquid partitioning. Next, the mixture was vigorously mixed for 5 min and extracted by ultrasonic for 20 min, followed by centrifuging at 6000 rpm for 5 min. After that, 15.0 mL supernatant was added into a heart-shaped flask (50-mL) and evaporated to dryness under reduced pressure. The evaporated residue was dissolved in 1.5 mL methanol/water solution and transferred into a centrifuge tube (5-mL) containing 300.0 mg MgSO_4_, 90.0 mg C_18_ sorbent, and 90.0 mg PSA sorbent for removing the residual water, sugar, and organic acid components. The mixture was vigorously shaken for 1 min and centrifuged at 8000 rpm for 5 min. Finally, the supernatant was filtered through a 0.20 μm filter before SERS analysis. The whole sample preparation process was repeated five times for each spiked level, a total of 30 spiked samples were prepared and assigned to the prediction set.

The blank apple matrix solution was prepared as in the above steps but without adding pymetrozine and carbendazim mixed solution. Pymetrozine and carbendazim matrix standard solutions were prepared by diluting their standard solutions with the blank matrix solution, the concentrations of pymetrozine and carbendazim in the matrix standard solutions were the same as those of the prediction set in Table S1. Pymetrozine and carbendazim matrix standard solutions (12 samples) were labeled as the calibration set.

### 2.5 SERS determination of pesticide solution

Prior to SERS measurement, a portion of 400.0 μL of Au@AgNPs was mixed with 100.0 μL of pesticide solution in a centrifuge tube (1.5-mL), and the mixture was stirred for 10 s, then 20.0 μL of NaOH solution (1 mol/L) was added to the tube and mixed for 10 s. The above solution was sucked into a capillary with an inner diameter of 1 mm and analyzed by a laser confocal microscopic Raman system (LabRAM HR, Horiba France SAS, Villeneuve, France). The system was calibrated by silicon wafer with a Raman shift of 520.66 cm^−1^. A pre-experiment was performed to determine the laser used in this study and acquisition time by using pymetrozine and carbendazim as probe molecules. The results of pre-experiment are shown in Fig. S2. Finally, a 633 laser was selected with an acquisition time set to 30 s and accumulated twice based on the comprehensive consideration of SERS intensity, instrument range, and continuous collection time of each spectrum. All Raman spectra were collected in the range of 400–1800 cm^−1^. Each sample was repeated three times, and the mean value was used for analysis.

### 2.6 LC-MS/MS analysis

The details of LC-MS/MS analysis are provided in the Supporting Information.

### 2.7 Data analysis

#### 2.7.1 Spectral pretreatment

Prior to chemometric analysis, the acquired spectra were first processed with smoothing by the LabSpec 6 software suite (Horiba France SAS, Villeneuve, France) to eliminate the interference of noise. To remove or eliminate the adverse effects of interference signals from different sources, the obtained spectra were further processed by two frequently used normalization methods including Multiplicative Scatter Correction (MSC) and Standard Normal Variate (SNV), and a common baseline correction method Savitzky-Golay (S-G) 2nd Derivation for comparison. MSC can enhance the correlation between the spectra data and measured values by eliminating the effect of scattering level differences on the spectra. SNV can center and scale individual spectra to remove slope variation, correct instrument noise, and background interference (Liland, Kohler, & Afseth, 2016). S-G 2nd derivation was used to reduce the influence of baseline drift of spectra. Both raw and processed spectra were used for building models for predicting pesticide contents.

#### 2.7.2 Chemometric models

It is generally believed that the SERS intensity of pesticides is linearly related to their concentration. Thus, linear algorithms are preferred for modeling. To obtain an optimal model for predicting pymetrozine and carbendazim contents, three frequently used linear regression algorithms including Multiple Linear Regression (MLR), Principal Component Regression (PCR), and Partial Least Squares Regression (PLSR) were employed to establish the regression models. Besides, Support Vector Machine Regression (SVMR) was chosen as a non-linear regression algorithm for comparison.

To validate the built models, the coefficient of determination (R^2^) and root mean square error (RMSE) were calculated and evaluated. Generally, the model with high R^2^, low RMSE, and small difference between RMSE of cross-validation (RMSECV) and RMSE of prediction (RMSEP) is considered reliable (Kutsanedzie et al., 2018). Residual predictive deviation (RPD) is the ratio of the standard deviation of reference values to RMSE. When the RPD value is 2–3 and greater than 3, the build model is considered to have good predictive performance and excellent predictive accuracy, respectively. If the RPD value is lower than 2, means the model is unreliable (Guo, Ni, & Kokot, 2016). Data analysis was achieved based on the Unscrambler X 10.4 software (Camo, Oslo, Norway).

## 3 Results and discussion

### 3.1 Characterization of Au@AgNPs

**Fig. 1:**
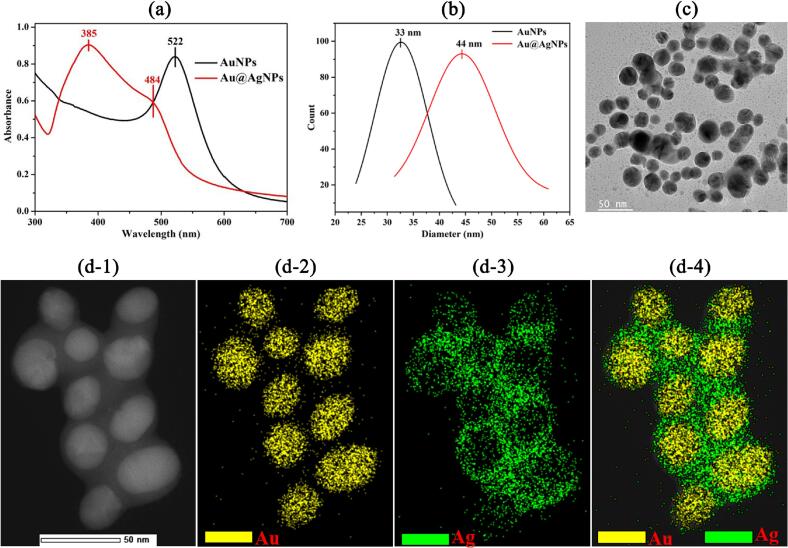
(a & b) UV–vis spectra and size distributions of AuNPs and Au@AgNPs; (c & d-1) TEM and HAADF-STEM images of Au@AgNPs; (d-2, 3, and 4) the HAADF-STEM-EDX elemental mapping of Au, Ag, and Au—Ag.

**Fig. 2:**
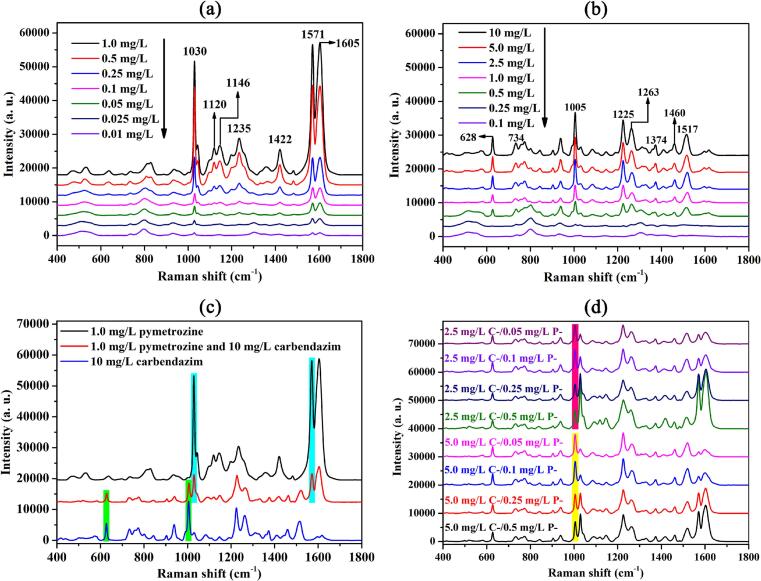
SERS spectra of different concentrations of (a) pymetrozine and (b) carbendazim; (c) SERS spectra of pymetrozine and carbendazim mixed and individual standard solutions at the same cncentrations; (d) SERS spectra of the mixed standard solutions with two fixed carbendazim concentrations (5.0 and 2.5 mg/L) but different pymetrozine concentrations (0.05–0.5 mg/L).

**Fig. 3:**
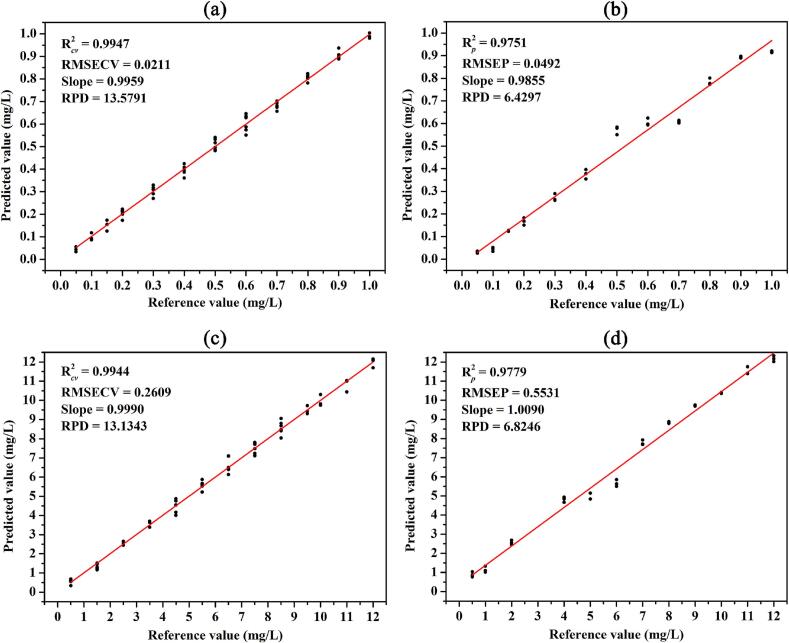
Scatter plot of the predicted value versus reference value for pymetrozine/carbendazim content of the calibration (a)/(c) and prediction (b)/(d) sets obtained by the SNV-PLSR model.

**Fig. 4:**
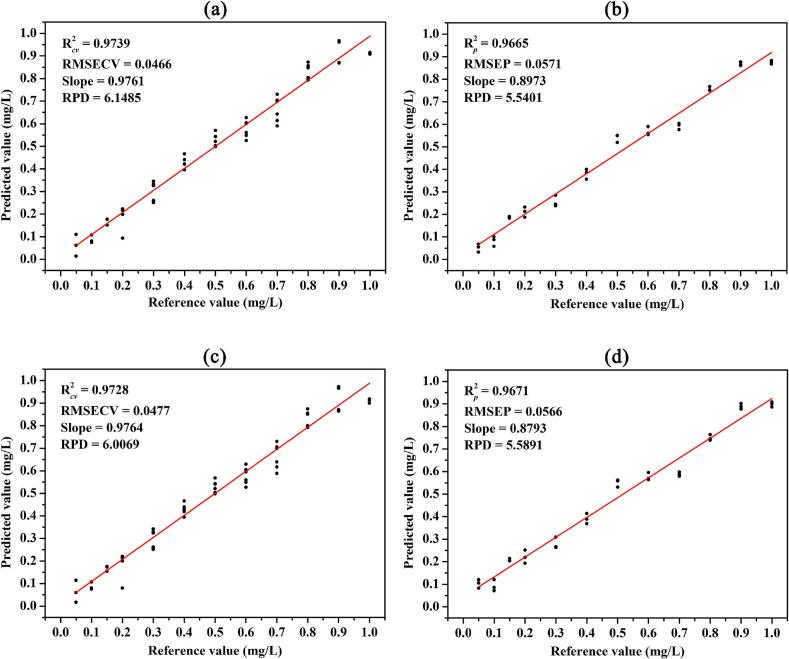
Scatter plot of the predicted value versus reference value for pymetrozine content of the calibration (a)/(c) and prediction (b)/(d) sets obtained by the SNV-PCR/SNV-PLSR model.

Au@AgNPs are obtained via a seed-mediated growth method by using AuNPs as a seed. Due to the presence of compatible crystalline lattices of the Au and Ag, AgNPs could grow on the surface of Au core to form Au@AgNPs with core-shell structure. Fig. 1a presents the UV–vis spectra of AuNPs and Au@AgNPs. A narrow band at 522 nm is observed in the UV–vis spectrum of AuNPs due to the surface plasmon resonance of AuNPs, while in the UV–vis spectrum of Au@AgNPs, two bands centered at 484 and 385 nm are observed, which is ascribed to overlapping and coupling plasmon resonance frequencies of Au core and Ag shell forming by the deposition of AgNPs on the Au surface, respectively. The results of DLS analysis for AuNPs and Au@AgNPs are presented in Fig. 1b, which show that the hydrodynamic diameter of the AuNPs is concentrated in the range of 29–38 nm, and it increased to 40–48 nm after the deposition of the silver shell (Au@AgNPs). Fig. 1c exhibits a transmission electron microscope (TEM) image of Au@AgNPs, it is revealed that Au@AgNPs is a core-shell structure nanoparticle including the center of Au core with an average diameter of 35 nm (the dark area) and the external surface of Ag shell with an average thickness of 10 nm (the bright color). In addition, to understand the distribution of Au and Ag elements more comprehensively, HAADF-STEM analysis and the corresponding EDS elemental mappings were performed. As shown in Fig. 1d-1, the core-shell architecture could also be observed from the HAADF-STEM image. The architecture of the Au@AgNPs was further confirmed by the EDS elemental mapping images. As shown in Fig. 1d-Fig. 2, Fig. 3, Fig. 4, the yellow and green hue represented the spatial distribution of Au and Ag element, respectively. It can be seen that the Ag element was distributed on the surface of Au core. These results had a similar trend to our previous studies (Guo et al., 2022; Pan, Guo, Lu, & Hu, 2021), indicating the effective synthesis of the Au@AgNPs.

### 3.2 Spectral features of pymetrozine and carbendazim in standard solutions

#### 3.2.1 Spectral analysis of pymetrozine and carbendazim in single standard solution

Prior to simultaneous detection, SERS spectra of pymetrozine and carbendazim were first collected from their single standard solutions with different concentrations. SERS spectra of pymetrozine and carbendazim with different concentrations are given in Fig. 2. As shown in Fig. 2a, the main characteristic peaks of pymetrozine can be observed at 1030, 1120, 1146, 1235, 1422, 1571, and 1605 cm^−1^. The results are consistent with previously reported studies (Guo et al., 2022; Li et al., 2023; Pan, Guo, Guo, et al., 2021; Xu et al., 2021; Xu et al., 2020). For carbendazim, the main characteristic peaks are located at 628, 734, 1005, 1225, 1263, 1374, 1460, and 1517 cm^−1^, as illustrated in Fig. 2b. Most of these peaks are similar to those reported in previous literature (Li et al., 2022; Strickland & Batt, 2009). The assignments for pymetrozine and carbendazim in the SERS spectra are summarized in Table S2. The SERS intensities of pymetrozine and carbendazim increased as their single concentration increased in a series of standard solutions, pymetrozine could be detected at a level of 0.01 mg/L, and carbendazim could be easily identified as low as 0.1 mg/L according to the most prominent peak of pymetrozine and carbendazim, respectively.

#### 3.2.2 Spectral analysis of pymetrozine and carbendazim in the mixed solution

The SERS spectrum of the mixed solution with 1.0 mg/L of pymetrozine and 10 mg/L of carbendazim is presented in Fig. 2c, and SERS spectra of their individual at the same concentrations are also given as a comparison. It can be seen that characteristic peaks of pymetrozine (such as 1030 and 1571 cm^−1^) and carbendazim (such as 628 and 1005 cm^−1^) in the mixed solution are almost not shifted. In addition, the different characteristic peaks of pymetrozine and carbendazim can be specifically distinguished and simultaneously detected in the SERS spectrum of the mixed solution. These results indicate that the method could be employed to accurately identify and quantify the two pesticides through a single SERS analysis (Ma et al., 2023; Yang et al., 2022). Moreover, an obvious phenomenon was observed that the SERS intensity of each pesticide in the mixed solution was significantly lower than those in their single solutions, which might be related to the competitive adsorption of pymetrozine and carbendazim on the surface of Au@AgNPs (Ma et al., 2023). Competitive adsorption reduced the total number of each pesticide molecule adsorbed onto Au@AgNPs surface, resulting in a decrease in the local concentration of each pesticide at the hotspots of Au@AgNPs, and finally leading to a decrease in their SERS intensity (Altun, Bond, Pronk, & Park, 2017).

Fig. 2d illustrates the SERS spectra of the mixed standard solutions with two fixed carbendazim concentrations (5.0 and 2.5 mg/L) but a range of different pymetrozine concentrations (0.05, 0.1, 0.25, and 0.5 mg/L), and a characteristic peak at 1005 cm^−1^ of carbendazim was marked in the figure. It could be observed that even though the carbendazim concentration was constant, the SERS intensity of carbendazim at 1005 cm^−1^ is variable, and the intensity of carbendazim mixed with a high concentration is lower than that mixed with a low concentration of pymetrozine. In addition, Fig. S3 displays SERS intensity at 1571 cm^−1^ from SERS spectra of different mixed solutions. As shown in Fig. S3, the concentration of pymetrozine was constant at 0.05, 0.1, 0.25, and 0.5 mg/L, respectively, and the concentrations of carbendazim were set to 0 (pymetrozine alone), 2.5, and 5.0 mg/L for comparison. It could be observed that when the concentration of pymetrozine was constant, SERS intensity at 1571 cm^−1^ related to pymetrozine decreased with the increase of carbendazim concentration, when the concentration of carbendazim was 5.0 mg/L, SERS intensity of pymetrozine was the lowest. The results further confirmed the competitive adsorption of pymetrozine and carbendazim on Au@AgNPs surface (Altun et al., 2017). Hence, it is unreasonable to utilize the linear correlation between the characteristic peak intensity and the pesticide concentration for quantitative assessment of a single pesticide in a mixed solution. This is due to potential variations in the characteristic peak intensity of the same pesticide within a mixed solution, resulting from its interaction with different pesticides or varying concentrations of the same pesticide. Therefore, it is necessary to use the spectra of the mixed solutions under the condition of competitive adsorption as a data set to improve the accuracy of the quantitative analysis.

### 3.3 Simultaneous determination of pymetrozine and carbendazim in the mixed solution by multivariate analysis

A total of 32 pymetrozine and carbendazim mixed solutions were prepared, and then 3 SERS spectra were collected for each mixed solution. Finally, a total of 96 SERS spectra over the range of 400–1800 cm^−1^ were obtained and divided into two groups: the calibration set comprised 60 spectra and the prediction set contained 36 spectra. These spectra were used for modeling analysis, the calibration set was applied to build the model and validate it, while the prediction set was applied to check the robustness and practicability of the newly built model.

Prior to modeling, all the obtained spectra were first preprocessed by MSC, SNV, and S-G 2nd derivation transformation. The raw and processed spectra of all samples are given in Fig. S4. Then, the PCR and PLSR were used to predict pymetrozine and carbendazim contents in the mixed solution based on the same spectral data. In addition, the SVMR model was built for comparison.

The results of PCR, PLSR, and SVMR models based on full-band SERS spectra with different spectral preprocessing methods to predict pymetrozine and carbendazim contents in the mixed solution are summarized in Table 1. In comparison with these three models, both PCR and PLSR models show better performance for pymetrozine and carbendazim content prediction as can be seen from the higher R^2^, lower RMSE, and small difference between RMSECV and RMSEP. In comparison with the three selected preprocessing methods, models based on the MSC and SNV processed spectra are significantly better than those of the original and S-G 2nd derivation processed spectra. After SNV pretreatment, the discrete degree between the raw and mean spectra was weakened by dividing standard deviation, which can effectively remove the variation generated by stochastic noise, instrument noise, background signal, and fluorescence interference, finally improving the model's accuracy. The MSC corrected spectra are obtained by subtracting every original spectral value by the additive effect and dividing by the multiplicative effect, which is beneficial to reduce the effects generated by background interference, ultimately improving the model's precision (Liland et al., 2016). In particular, SNV-PLSR model is the optimal model for both pymetrozine and carbendazim contents prediction, with R2 *cv* of 0.9947, RMSECV of 0.0211 mg/L, R2 *p* of 0.9751 and RMSEP of 0.0492 mg/L for pymetrozine, and R2 *cv* of 0.9944, RMSECV of 0.2609 mg/L, R2 *p* of 0.9779, and RMSEP of 0.5531 mg/L for carbendazim.

**Table 1:** Comparison of the predictive performance for pesticide content of various models based on different processing spectra.

Pesticide	Model	Spectra	R2 *cv*	RMSECV (mg/L)	R2 *p*	RMSEP (mg/L)
Pymetrozine	PCR	Raw	0.9551	0.0620	0.8737	0.1109
		MSC	0.9757	0.0449	0.9509	0.0691
		SNV	0.9798	0.0410	0.9743	0.0500
		S-G 2nd Der	0.9471	0.0663	0.6000	0.1973
	PLSR	Raw	0.9633	0.0557	0.8670	0.1138
		MSC	0.9911	0.0272	0.9501	0.0697
		**SNV**	**0.9947**	**0.0211**	**0.9751**	**0.0492**
		S-G 2nd Der	0.9612	0.0567	0.7268	0.1630
	SVMR	Raw	0.7515	0.1487	0.6795	0.1247
		MSC	0.9833	0.0388	0.9419	0.0699
		SNV	0.9829	0.0394	0.9555	0.0630
		S-G 2nd Der	0.9491	0.0735	0.5357	0.1419
Carbendazim	PCR	Raw	0.9580	0.7123	0.8792	1.2938
		MSC	0.9868	0.3962	0.9516	0.8189
		SNV	0.9904	0.3358	0.9765	0.5711
		S-G 2nd Der	0.9521	0.7590	0.7019	2.0323
	PLSR	Raw	0.9706	0.5937	0.8670	1.3673
		MSC	0.9931	0.2880	0.9723	0.6199
		**SNV**	**0.9944**	**0.2609**	**0.9779**	**0.5531**
		S-G 2nd Der	0.9707	0.5922	0.7404	1.8964
	SVMR	Raw	0.7789	1.7228	0.9250	1.5433
		MSC	0.9827	0.5134	0.9563	0.7804
		SNV	0.9815	0.5076	0.9636	0.7564
		S-G 2nd Der	0.9654	0.8828	0.8180	1.6826

Fig. 3a and b show scatter plots of the predicted value versus reference value for pymetrozine content of the calibration and prediction sets obtained by the SNV-PLSR model. It can be observed that the SNV-PLSR model has a good linear correlation between the predicted and spiked pymetrozine concentrations in the mixed solutions (R2 *cv* = 0.9947 and R2 *p* = 0.9751), and the RPD of the validation model and prediction model was 13.5791 and 6.4297, respectively, which indicates the predictive accuracy of the built model was excellent. Scatter plots of the predicted value versus reference value for carbendazim content of the calibration and prediction sets obtained by the SNV-PLSR model are presented in Fig. 3c and d. The SNV-PLSR model shows an excellent correlation between the predicted and spiked carbendazim concentrations according to the R^2^ value of the calibration and prediction set was 0.9944 and 0.9779, respectively. The RPD of the validation model and prediction model was 13.1343 and 6.8246, respectively. The SNV-PLSR model is considered to have excellent predictive accuracy for both pymetrozine and carbendazim contents prediction as the RPD was greater than 6 (Yan et al., 2023).

Although the built models based on the full-band SERS spectra show satisfactory performance for pymetrozine and carbendazim content prediction, the dimensions of the input data are high in data volume. The full-band SERS spectrum would contain numerous irrelevant variables that are not related to pesticide content. These irrelevant variables can not only reduce the prediction accuracy, but also increase the computational resources and computational time (Li et al., 2022; Zhu et al., 2021). The optimal variables were selected and employed for modeling is one of the best strategies to solve the problem of large datasets. In this study, the optimal variables of pymetrozine and cabbendazim were manually selected based on the characteristic peaks in their SERS spectra. Then prediction models based on the selected variables were established for comparison. Four different multivariate analysis algorithms including MLR, PCR, PLSR, and SVMR were selected for modeling. The predicted performances of the built models are summarized in Table S3. As shown in Table S3, based on the characteristic peaks of pymetrozine, PLSR and PCR models have better predictive ability (high R^2^ and low RMSE values, and small difference between RMSECV and RMSEP) than the other two models. Fig. 4a, b and c, d illustrate scatter plots of the predicted value versus reference value for pymetrozine content of the calibration and prediction sets obtained by the SNV-PCR and SNV-PLSR models. Both SNV-PCR model (R2 *cv* of 0.9739, RMSECV of 0.0466 mg/L, RPD of 6.1485, R2 *p* of 0.9665, RMSEP of 0.0571, RPD of 5.5401) and SNV-PLSR model (R2 *cv* of 0.9728, RMSECV of 0.0477 mg/L, RPD of 6.0069, R2 *p* of 0.9671, RMSEP of 0.0566, RPD of 5.5891) show satisfied prediction result. For carbendazim content prediction, Fig. S5b and d show a good linear relationship in the PCR model (R2 *p* = 0.9232, RMSEP = 1.0317, and RPD = 3.6587) and PLSR model (R2 *p* = 0.9231, RMSEP = 1.0319, and RPD = 3.6580) between the reference and predicted carbendazim contents in the mixed solution. In summary, predictive performances for both pymetrozine and carbendazim contents based on full-band SERS spectra were better than those of the characteristic peaks. Although the predictive performances of both PCR and PLSR models based on the characteristic peaks were not as good as those of the full-band spectra, the number of variables used for building a reliable model was remarkably reduced to 7 or 8 peaks, which was crucial for online rapid detection using SERS technology in the future (Yan et al., 2023). These findings validate that the SERS technique in combination with chemometric methods is a reliable and effective method for simultaneous detection and quantification of pymetrozine and carbendazim in their mixed solutions.

### 3.4 The PLSR model for predicting pymetrozine and carbendazim contents in apple

**Fig. 5:**
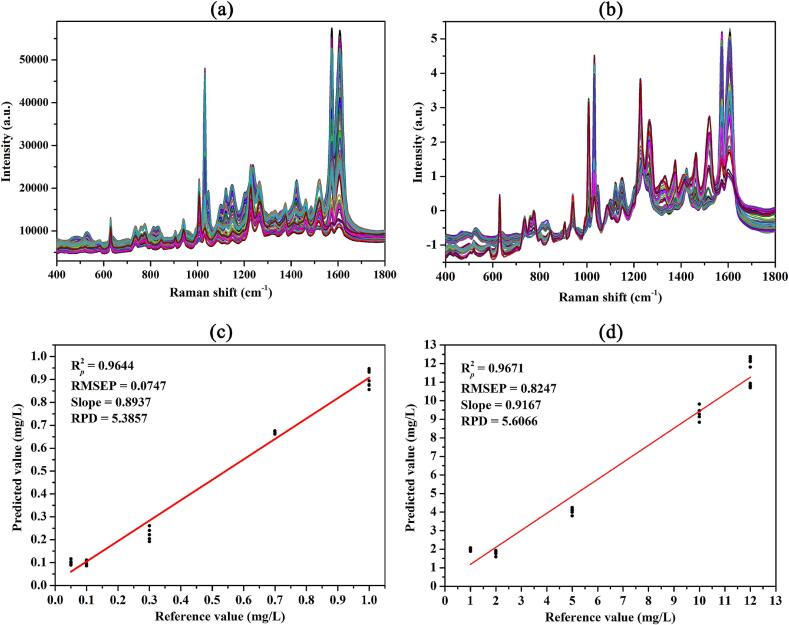
The raw (a) and SNV processed (b) SERS spectra of all samples; Scatter plot of the predicted value versus reference value for pymetrozine (c) and carbendazim (d) contents of the prediction set obtained by the SNV-PLSR model.

The raw and SNV processed spectra of pymetrozine and carbendazim in the apple extract are presented in Fig. 5a and b. As shown in Fig. 5a, the characteristic peaks of the apple extracts contaminated with pymetrozine and carbendazim were similar to those of pymetrozine and carbendazim mixed solution, while SERS intensities of these peaks were slightly lower than those in the mixed solution at the same concentration. Apple is a common fruit that contains many complex components, such as sugars, organic acids, pigments, etc. These non-target components exert a negative impact on the enhancement effect, leading to varying degrees of interference with the SERS signals of pymetrozine and carbendazim. These components impede the adsorption of target molecules onto the surface of SERS active substrate. Furthermore, organic acids present in apple matrix may induce alterations in pH value in the system, ultimately impacting SERS analysis. Additionally, certain non-target components may generate Raman signals that disrupt the spectra of target molecules, thereby compromising the accuracy of SERS analysis (Alsammarraie et al., 2018; Fan, Lai, Rasco, & Huang, 2014; Song, Qiu, Huang, Wang, & Lai, 2023). In addition, the SNV processed spectra of apple extract (Fig. 5b) were also similar to those of the mixed solution (Fig. S4c). After evaluating the performance of four models based on spectra obtained from mixed solutions, the PLSR model was ultimately chosen for predicting concentrations of pymetrozine and carbendazim residues in apple samples. Table S4 shows the PLSR analysis results based on the SERS spectra of apple extract spiked with a series of pymetrozine and carbendazim mixed solutions. Fig. 5c and d illustrate the PLSR model for the prediction of pymetrozine and carbendazim in apple based on the calibration curve of reference and predicted concentrations of the two pesticides. An excellent relationship between the reference and predicted pymetrozine and carbendazim concentrations in apple samples was established as shown in Fig. 5c and d (R2 *p* = 0.9644, RMSEP = 0.0747 mg/L, slope = 0.8937 and R2 *p* = 0.9671, RMSEP = 0.8247 mg/L, slope = 0.9167, respectively). RPD value was 5.3857 and 5.6066 for pymetrozine and carbendazim residues in apple, respectively, indicating robust PLSR models were built. To evaluate the applicability of the proposed SERS method for predicting unknown samples, the spiked and predicted results of the prediction set of the PLSR model are applied for recovery analysis. As shown in Table 2, the proposed method achieved the recovery ranged from 74.65 % to 100.39 % with RSD lower than 12.32 % and varied from 80.94 % to 95.65 % with RSD lower than 7.20 % for pymetrozine and carbendazim, respectively, indicating the developed method based on PLSR model has high accuracy and precision to predict pymetrozine and carbendazim contents in apple. According to the system's lowest detection concentration, the LOD of the method for pymetrozine and carbendazim was 0.03 and 0.3 mg/L, respectively (Li et al., 2020). These results demonstrate that it is an effective approach to use SERS technique coupled with the PLSR model to detect both pymetrozine and carbendazim residues in apple. In this study, the PLSR model showed the strongest predictive ability for the content of pymetrozine and carbendazim. However, a major drawback of PLSR is that it cannot provide quantitative explanations for the complex relationship between sample properties and spectra. Therefore, future work should avoid using PLSR to handle large datasets with high variability. Another drawback of PLSR is the difficulty of transferring the prediction model from one sensor to another (Clingensmith & Grunwald, 2022; Li et al., 2012). SERS technology has great potential in pesticide analysis, however, the complex non-target components in food make the application of SERS technology in food pesticide residue analysis very difficult. To eliminate the negative impact of food matrix effects on SERS analysis, a series of pretreatment methods are needed to process the samples to exclude interfering components, concentrate the analyte molecules, and finally improve the detection efficiency.

**Table 2:** Quantification of pymetrozine and carbendazim residues in apple by the proposed method.

Pesticide	Spiked value (mg/L)	Predicted value (mg/L)	Recovery (%)	RSD (%)
Pymetrozine	0.1	0.1004 ± 0.0104	100.39	10.37
	0.3	0.2239 ± 0.0276	74.65	12.32
	0.7	0.6668 ± 0.0055	95.25	0.82
	1.0	0.9064 ± 0.0345	90.64	3.81
Carbendazim	2	1.7868 ± 0.1287	89.34	7.20
	5	4.0471 ± 0.1723	80.94	4.26
	10	9.3048 ± 0.3650	93.05	3.92
	12	11.4781 ± 0.7267	95.65	6.33

### 3.5 Comparison with LC-MS/MS method

The LC-MS/MS method was used to validate the reliability of SERS method for detecting pymetrozine and carbendazim residues in apple. The chromatogram and standard curves of pymetrozine and carbendazim standard solution are shown in Fig. S6a, b, and c. The retention time of pymetrozine and carbendazim was 2.70 and 3.76 min, respectively. The results of recovery experiment for detecting pymetrozine and carbendazim in apple by LC-MS/MS were summarized in Table S5. As shown in Table S5, the recovery for pymetrozine in apple ranged from 88.65 % to 104.46 % with RSD below 5.66, and it ranged from 95.29 % to 105.28 % with RSD below 9.09 for carbendazim, indicating the LC-MS/MS method exhibits high accuracy and precision. A comparison between the results of LC-MS/MS and SERS methods was conducted. The results are presented in Fig. S6d, which showed a good correlation between the two methods with *y* = 0.9033 *x* - 0.0469 and *R*^2^ = 0.9956. Hence, SERS is a reliable method for detecting pymetrozine and carbendazim residues in apple.

## 4 Conclusions

In this study, SERS technique combined with chemometric methods was applied to simultaneously detect pymetrozine and carbendazim contents in the mixed standard solutions and apple. To reduce the influence of competitive adsorption of pymetrozine and carbendazim on Au@AgNPs surface and improve the accuracy and robustness of the built model, a series of SERS spectra were acquired from pymetrozine and carbendazim mixed solutions with various concentration ratios, which were then applied for modeling. A total of 4 regression models were built and compared using both raw and processed spectra, as well as optimal variables. The PLSR model based on full-band SNV processed spectra yielded the optimal result for pymetrozine and carbendazim contents prediction, with R2 *p* of 0.9751 and 0.9779, RMSEP of 0.0492 and 0.5531 mg/L, RPD of 6.4297 and 6.8246, respectively. This model also achieved the optimal performance for detecting pymetrozine and carbendazim residues in apple (R2 p of 0.9644 and 0.9671, RMSEP of 0.0747 and 0.8247 mg/L), which was confirmed by the high RPD value of 5.3857 and 5.6066 for pymetrozine and carbendazim, respectively. The recovery for pymetrozine and carbendazim in apple was 74.65 %–100.39 % and 80.94 %–95.65 %, and precision tests showed satisfactory results with RSD lower than 12.32 % and 7.20 %, respectively. Meanwhile, the obtained results by SERS method were almost similar to LC-MS/MS results. These results indicate that SERS technology coupled with chemometric analysis is a reliable method for simultaneous detection of pymetrozine and carbendazim in apple, providing a new way for simultaneous determination of mixed pesticide residues in foodstuffs.

## CRediT authorship contribution statement

**Ting-feng Shi:** Writing – original draft, Visualization, Validation, Methodology, Investigation, Formal analysis, Data curation. **Ting-tiao Pan:** Writing – review & editing, Supervision, Software, Project administration, Funding acquisition, Conceptualization. **Ping Lu:** Writing – review & editing, Resources.

## Declaration of competing interest

The authors declare that they have no known competing financial interests or personal relationships that could have appeared to influence the work reported in this paper.

## Data Availability

Data will be made available on request.

## Appendix A Supplementary data

Supplementary material Image 1
