# Supplementary material for: Rapid and simultaneous determination of mixed pesticide residues in apple using SERS coupled with multivariate analysis
Source: Food Chem X. 2024 Nov 1;24:101954. doi: 10.1016/j.fochx.2024.101954 (PMC11582432; doi:10.1016/j.fochx.2024.101954)
Supplement: Supplementary file 1 — Supplementary material [file mmc1.docx]

**Supporting Information**

**Rapid and simultaneous determination of mixed pesticide residues in apple using SERS coupled with multivariate analysis**

Ting-feng Shi^a^, Ting-tiao Pan^a,b^[[1]](#footnote-1)^*^, Ping Lu ^a*^

^a^National Key Laboratory of Green Pesticide, Key Laboratory of Green Pesticide and Agricultural Bioengineering, Ministry of Education, Center for R&D of Fine Chemicals of Guizhou University, Guiyang, 550025, China

^b^the Key Laboratory of Environmental Pollution Monitoring and Disease Control, Ministry of Education, Guizhou Medical University, Guiyang, 550025, China

2.6 LC-MS/MS analysis

The LC-MS/MS method was used as a reference method to determine the amounts of pymetrozine and carbendazim residues in apple. LC-MS/MS analysis was performed by an AB Sciex 4500 Q Trap LC-MS/MS system (AB SCIEX, USA) equipped with an electrospray ionization source. Chromatographic column (JADE-PAK UP-C18, 100 × 2.1 mm, 1.8 µm) used in this study was obtained from Guangzhou Taiwei Biotechnology Co., LTD (Guangzhou, China). The column temperature was maintained at 35 ^o^C. The mobile phase consisted of water (A) and methanol (B), at a flow rate of 0.3 mL/min in the gradient mode. The composition of the mobile phase varied as follows: 0-0.20 min: 20% B; 0.20-3.80 min: 90% B, 3.80-5.00 min: 90% B; 5.00-5.50 min: 20% B, the total analysis time was 5.5 min. The injection volume was 2 µL. Positive MRM mode was used to detect samples. The parameters for the MS/MS system were as follows: ion spray voltage was 5500 V, ion source temperature was 600 °C, curtain gas was 30 psi, ion source gas 1 was 70 psi, and gas 2 was 60 psi. Other MS/MS parameters of pymetrozine and carbendazim were summarized in Table S6.


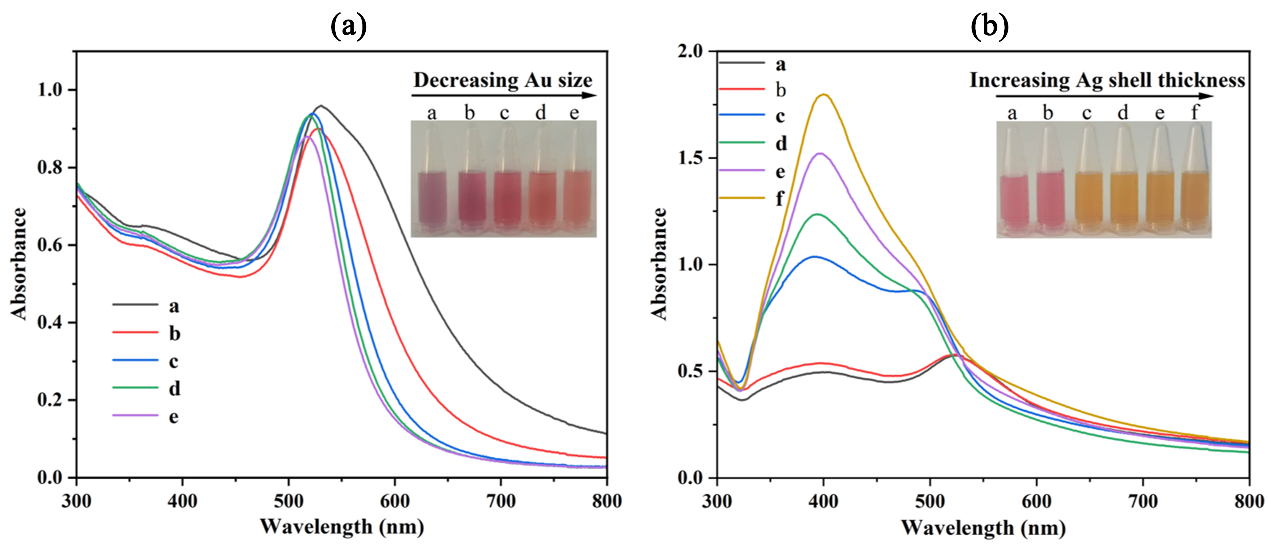


**Fig. S1** The UV-vis spectra of (a) different sizes of AuNPs and (b) varying Ag shell thicknesses of Au@AgNPs, the insert graphs display the corresponding colloidal solutions of different sizes of AuNPs and varying Ag shell thicknesses Au@AgNPs


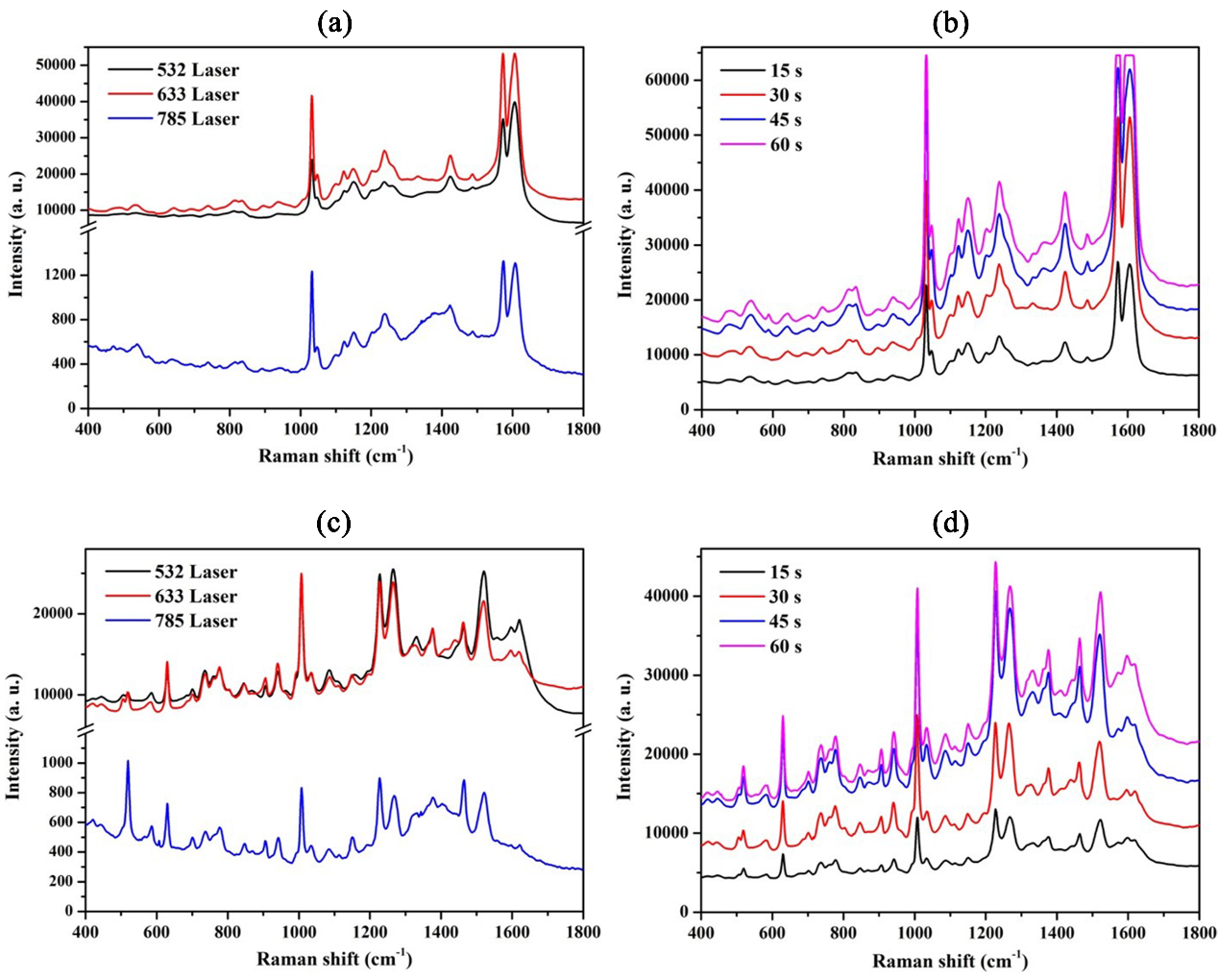


**Fig. S2** The effects of different lasers and acquisition time on SERS signals of (a & b) pymetrozine and (c & d) carbendazim


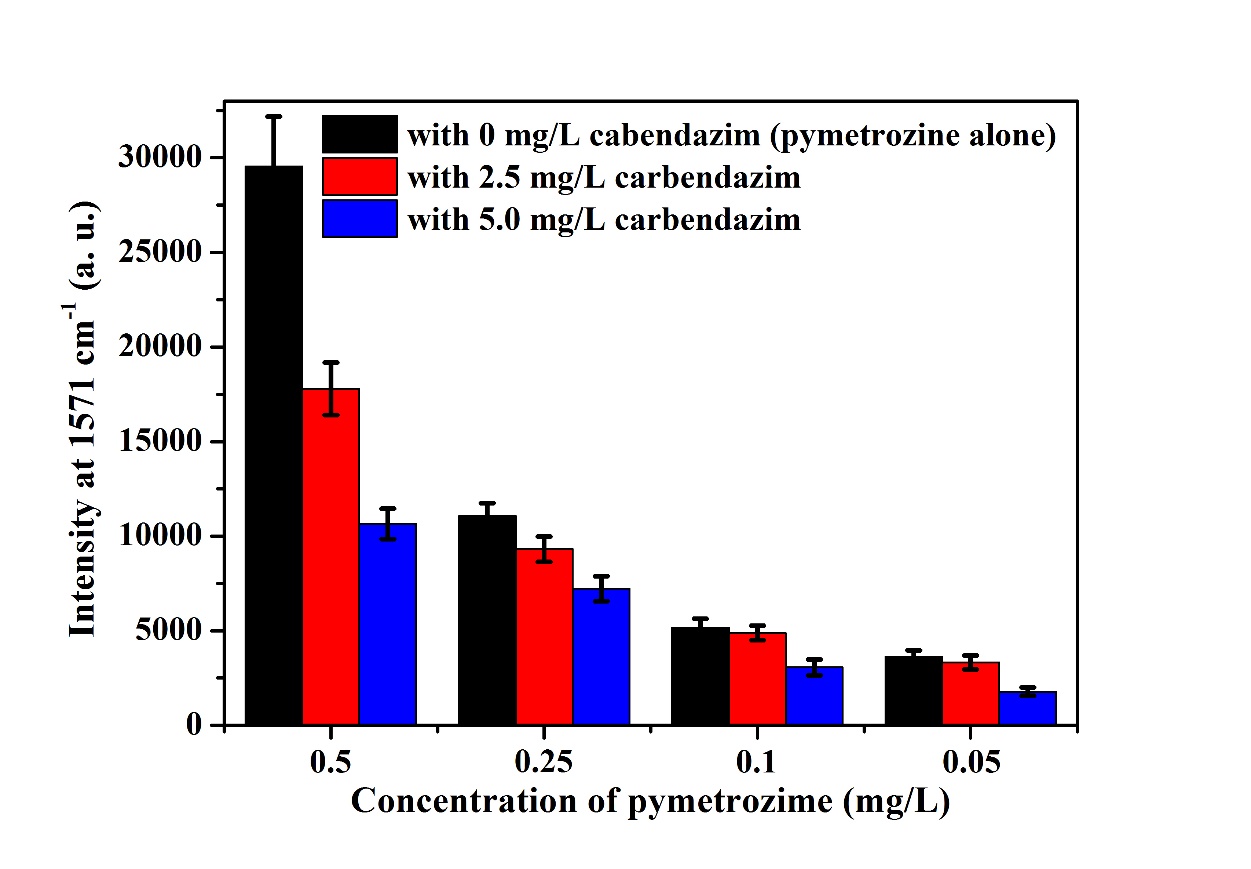


**Fig. S3** SERS intensity at 1571 cm^-1^ from SERS spectra of different mixed solutions


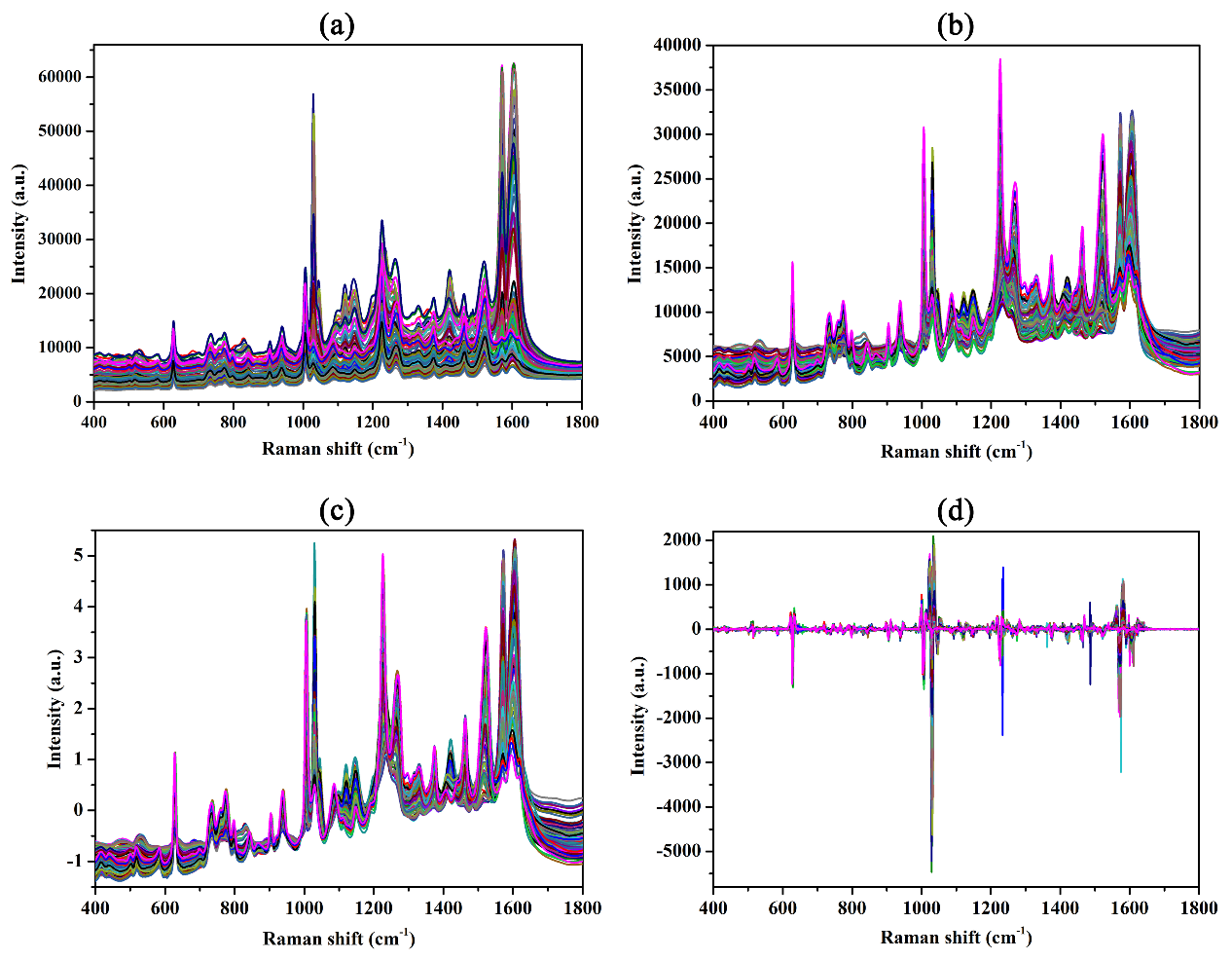


**Fig. S4** The raw (a) and MSC (b), SNV (c), and S-G 2^nd^ derivative (d) processed SERS spectra of all samples


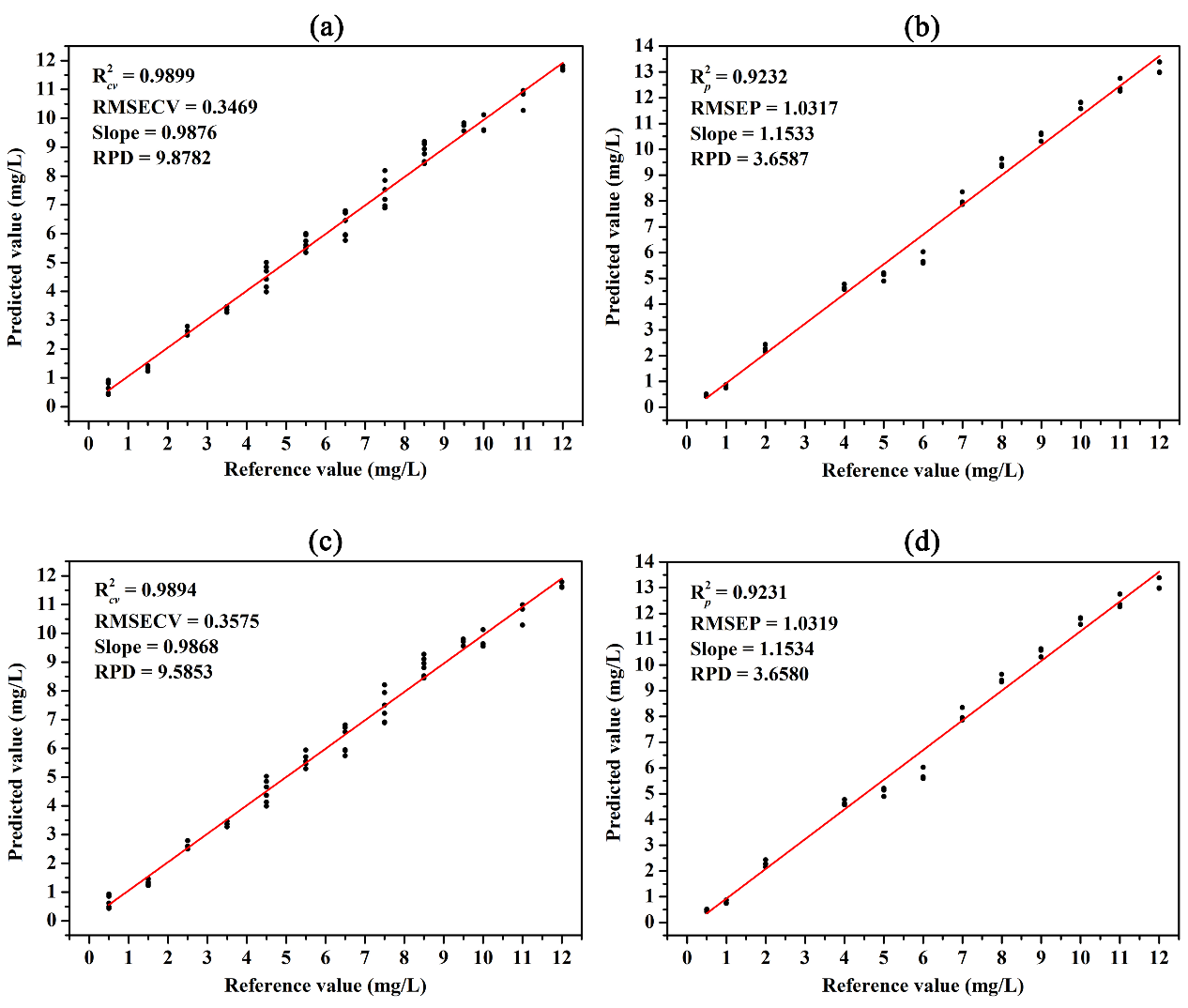


**Fig. S5** Scatter plot of the predicted value versus reference value for carbendazim content of the calibration (a)/(c) and prediction (b)/(d) sets obtained by the SNV-PCR/SNV-PLSR model


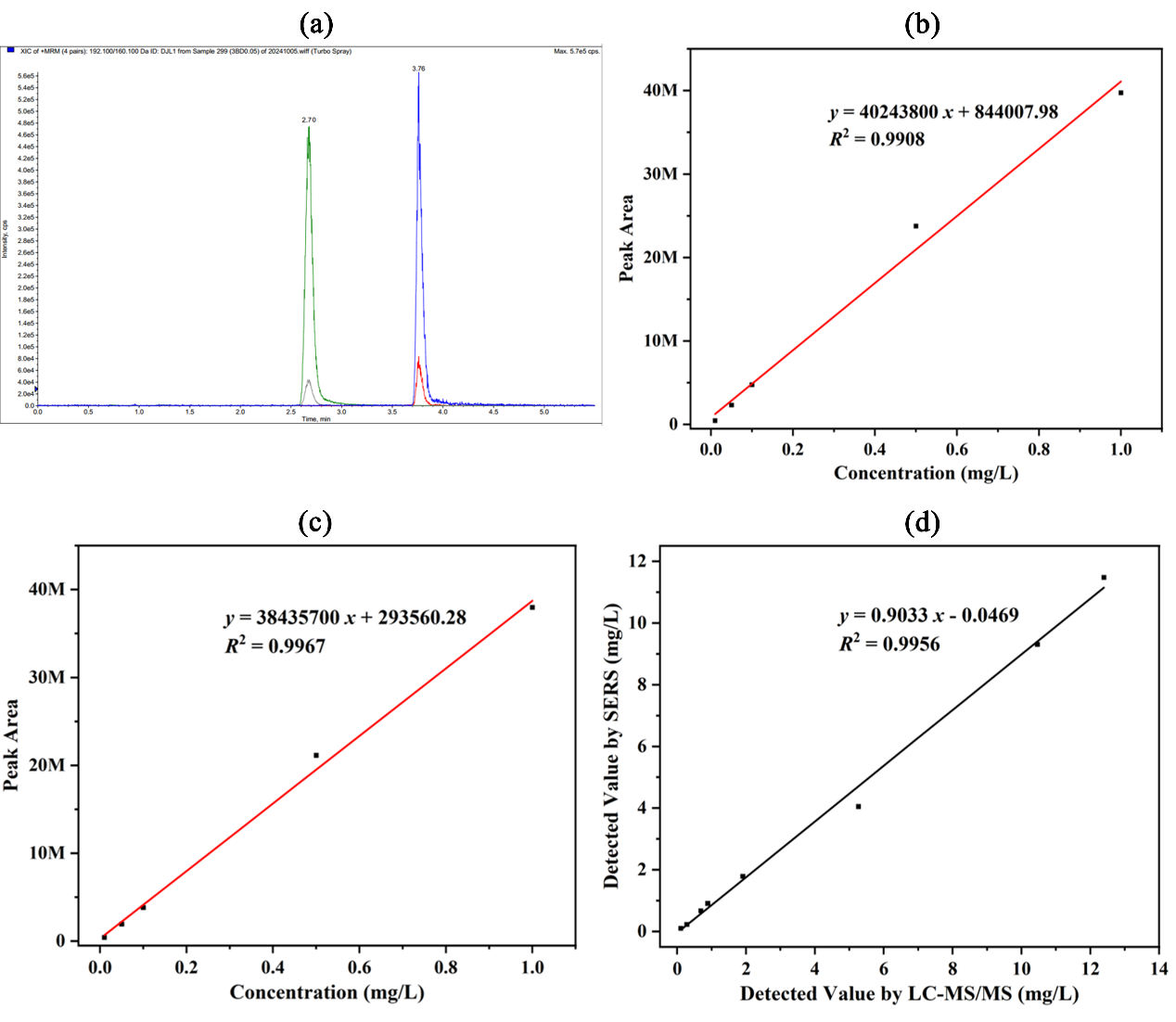


**Fig. S6** The chromatogram (a) and the standard curves of pymetrozine (b) and carbendazim (c) standard solution; the correlation (d) between the LC-MS/MS and SERS methods

**Table S1 Composition of the mixed solution in calibration set and prediction set**

| **Calibration set** | | | | | | **Prediction set** | | |
| --- | --- | --- | --- | --- | --- | --- | --- | --- |
| **No.** | **A** | **B** | **No.** | **A** | **B** | **No.** | **A** | **B** |
| **1** | 1.0 | 0.5 | **11** | 0.5 | 6.5 | **1** | 1.0 | 0.5 |
| **2** | 0.9 | 0.5 | **12** | 0.4 | 6.5 | **2** | 0.9 | 1.0 |
| **3** | 0.9 | 1.5 | **13** | 0.4 | 7.5 | **3** | 0.8 | 2.0 |
| **4** | 0.8 | 1.5 | **14** | 0.3 | 7.5 | **4** | 0.7 | 4.0 |
| **5** | 0.8 | 2.5 | **15** | 0.3 | 8.5 | **5** | 0.6 | 5.0 |
| **6** | 0.7 | 3.5 | **16** | 0.2 | 8.5 | **6** | 0.5 | 6.0 |
| **7** | 0.7 | 4.5 | **17** | 0.2 | 9.5 | **7** | 0.4 | 7.0 |
| **8** | 0.6 | 4.5 | **18** | 0.15 | 10 | **8** | 0.3 | 8.0 |
| **9** | 0.6 | 5.5 | **19** | 0.1 | 11 | **9** | 0.2 | 9.0 |
| **10** | 0.5 | 5.5 | **20** | 0.05 | 12 | **10** | 0.15 | 10 |
|  |  |  |  |  |  | **11** | 0.1 | 11 |
|  |  |  |  |  |  | **12** | 0.05 | 12 |

**A: the concentration of pymetrozine in the mixed solutions (mg/L)**

**B: the concentration of carbendazim in the mixed solutions (mg/L)**

**Table S2 The assignments for pymetrozine and carbendazim in SERS spectra**

| **Pesticide** | **Raman shift (cm^-1^)** | **Assignment** |
| --- | --- | --- |
| Pymetrozine | 1030 | υ (C-C) in ring and υ (C-N) in ring |
|  | 1120 | υ (N-N) |
|  | 1146 | υ (N-N) and ρ (C-H) |
|  | 1235 | δ (C-C-H) |
|  | 1422 | δ (C-H) |
|  | 1571 | υ (C=O) and ρ (C-H) |
|  | 1605 | υ (C-N), υ (C-C) in ring, and δ (C-C-H) |
| Carbendazim | 628 | δ (C-C-C) |
|  | 734 | δ (C-H) in ring |
|  | 1005 | υ (C-O) and υ (C=O) |
|  | 1225 | δ (N-H) |
|  | 1263 | δ (C-H) |
|  | 1374 | υ (C-N) |
|  | 1460 | ρ (C-H) |
|  | 1517 | υ (C-C) |

**Abbreviation:** υ: stretching; δ: bending; ρ: vibration

**Table S3 Comparison of the predictive performance for pesticide content of various models based on the characteristic peaks after different treatments**

| **Pesticide** | **Model** | **Preprocessing** | **R2 *cv*** | **RMSECV**  **(mg/L)** | **R2 *p*** | **RMSEP**  **(mg/L)** |
| --- | --- | --- | --- | --- | --- | --- |
| Pymetrozine | MLR | Raw | 0.9082 | 0.0868 | 0.6154 | 0.3120 |
|  |  | MSC | 0.9632 | 0.0550 | 0.9535 | 0.0767 |
|  |  | **SNV** | 0.9665 | 0.0525 | 0.9612 | 0.0601 |
|  |  | S-G 2^nd^ Der | 0.6300 | 0.1743 | 0.6011 | 0.1597 |
|  | PCR | Raw | 0.9146 | 0.0840 | 0.9033 | 0.0970 |
|  |  | MSC | 0.9696 | 0.0505 | 0.9396 | 0.0767 |
|  |  | SNV | 0.9739 | 0.0466 | 0.9665 | 0.0571 |
|  |  | S-G 2^nd^ Der | 0.7201 | 0.1546 | 0.7687 | 0.1500 |
|  | PLSR | Raw | 0.9269 | 0.0789 | 0.8760 | 0.1098 |
|  |  | MSC | 0.9694 | 0.0510 | 0.9396 | 0.0767 |
|  |  | **SNV** | **0.9728** | **0.0477** | **0.9671** | **0.0566** |
|  |  | S-G 2^nd^ Der | 0.7105 | 0.1552 | 0.7805 | 0.1461 |
|  | SVMR | Raw | 0.8625 | 0.1088 | 0.6812 | 0.1497 |
|  |  | MSC | 0.9768 | 0.0445 | NA | 0.2060 |
|  |  | SNV | 0.9770 | 0.0434 | 0.9816 | 0.0531 |
|  |  | S-G 2^nd^ Der | 0.8874 | 0.0980 | 0.7421 | 0.1424 |
| Carbendazim | MLR | Raw | 0.9034 | 1.0652 | 0.6232 | 3.7504 |
|  |  | MSC | 0.9871 | 0.3893 | 0.9842 | 5.6238 |
|  |  | SNV | 0.9889 | 0.3613 | 0.9890 | 1.0317 |
|  |  | S-G 2^nd^ Der | 0.6442 | 2.0439 | 0.03548 | 3.6816 |
|  | PCR | Raw | 0.8766 | 1.2237 | 0.8546 | 1.4193 |
|  |  | MSC | 0.9485 | 0.8742 | 0.7316 | 1.9283 |
|  |  | SNV | 0.9899 | 0.3469 | 0.9232 | 1.0317 |
|  |  | S-G 2^nd^ Der | 0.6765 | 1.9796 | 0.3277 | 3.0517 |
|  | PLSR | Raw | 0.8979 | 1.1160 | 0.8528 | 1.4282 |
|  |  | MSC | 0.9877 | 0.3823 | 0.9454 | 3.7225 |
|  |  | **SNV** | **0.9894** | **0.3575** | **0.9231** | **1.0319** |
|  |  | S-G 2^nd^ Der | 0.6167 | 2.1361 | 0.1871 | 3.3557 |
|  | SVMR | Raw | 0.7442 | 1.8647 | 0.7542 | 1.6992 |
|  |  | MSC | 0.9717 | 0.5875 | NA | 3.1689 |
|  |  | SNV | 0.9744 | 0.5511 | 0.9404 | 1.2362 |
|  |  | S-G 2^nd^ Der | 0.6870 | 1.9958 | NA | 4.0366 |

**Table S4 Comparison of the predictive performance for pesticide content of PLSR model based on different variables**

| **Pesticide** | **Variable** | ***R*2 cv** | **RMSECV**  **(mg/L)** | ***R*2 p** | **RMSEP**  **(mg/L)** | **RPD** |
| --- | --- | --- | --- | --- | --- | --- |
| Pymetrozine | Raw-F | 0.9938 | 0.0246 | 0.8519 | 0.1522 | 2.6433 |
|  | Raw-C | 0.9484 | 0.0721 | 0.9128 | 0.1168 | 3.4444 |
|  | **SNV-F** | **0.9965** | **0.0195** | **0.9644** | **0.0747** | **5.3857** |
|  | SNV-C | 0.9760 | 0.0517 | 0.8934 | 0.1291 | 3.1163 |
| Carbendazim | Raw-F | 0.9945 | 0.2748 | 0.9588 | 0.9232 | 5.0084 |
|  | Raw-C | 0.9513 | 0.8501 | 0.3846 | 3.5664 | 1.2965 |
|  | **SNV-F** | **0.9966** | **0.2228** | **0.9671** | **0.8247** | **5.6066** |
|  | SNV-C | 0.9580 | 0.7699 | 0.9299 | 1.2033 | 3.8426 |

Raw-F: Full-band of raw spectra

Raw-C: Characteristic peaks of raw spectra

SNV-F: Full-band of SNV spectra

SNV-C: Characteristic peaks of SNV spectra

**Table S5 Quantification of pymetrozine and carbendazim residues in apple by the LC-MS/MS method**

| **Pesticide** | **Spiked value**  **(mg/L)** | **Detected value**  **(mg/L)** | **Recovery**  **(%)** | **RSD**  **(%)** |
| --- | --- | --- | --- | --- |
| Pymetrozine | 0.1 | 0.1044±0.0008 | 104.46 | 0.85 |
|  | 0.3 | 0.2794±0.0158 | 93.15 | 5.66 |
|  | 0.7 | 0.6862±0.0205 | 98.03 | 2.99 |
|  | 1.0 | 0.8864±0.0120 | 88.65 | 2.25 |
| Carbendazim | 2 | 1.9057±0.0087 | 95.29 | 9.09 |
|  | 5 | 5.2640±0.0230 | 105.28 | 8.74 |
|  | 10 | 10.4623±0.0250 | 104.62 | 4.77 |
|  | 12 | 12.3928±0.0358 | 103.27 | 5.78 |

Table S6 **Retention time and MS/MS parameters of** **pymetrozine and carbendazim**

| **Pesticide** | **Retention time (min)** | **Precursor ion (m/z)** | **Product ion (m/z)** | **Declustering potential (V)** | **Collision energy (V)** | **Cell exit potential (V)** |
| --- | --- | --- | --- | --- | --- | --- |
| Pymetrozine | 2.70 | 217.900 | 105.100* | 40.00 | 26.12/54.94 | 5.00 |
|  |  |  | 79.000 |  |  |  |
| Carbendazim | 3.76 | 192.100 | 160.100* | 30.00 | 25.00/41.00 | 3.00 |
|  |  |  | 132.100 |  |  |  |

Note: * quantitative ion

1. * Corresponding author, E-mail: pantingtiaos@163.com (T.-t. Pan); plu@gzu.edu.cn (P. Lu) [↑](#footnote-ref-1)
